# Analysis of transmitted drug resistance, resistance mutations and future antiretroviral efficacy in HIV-1 subtype F infected-patients prior to therapy

**DOI:** 10.1186/1758-2652-13-S4-P126

**Published:** 2010-11-08

**Authors:** LSC Manolescu, CM Sultana, C Vagu, A Temereanca, SM Ruta

**Affiliations:** 1 Institute of Virology, "Stefan S. Nicolau", Bucharest, Romania; 2 “Carol Davila” University of Medicine and Pharmacy, Bucharest, Romania; 3 Institute of Virology "Stefan S. Nicolau", Bucharest, Romania

## Background

Studies have shown that transmitted drug resistance mutations involving major antiretroviral classes appear to persist for months, even years, after infection. Efficacy of antiretroviral treatment in recently infected individuals imply resistance testing prior to initialize treatment. The prevalence of drug resistance mutations in patients newly infected with HIV-1 is high throughout the world and little is known about transmission of resistant virus in non B subtypes. In Romania from 1992 to 2009, analysis of the relative incidence of different HIV-1 circulating strains revealed a stable profile with high prevalence of subtype - F, in both long term survivors and recently infected adults.

## Objectives

To study the prevalence of transmitted drug resistance and asses the type of mutations fond in F subtype recently diagnosed and naïve patients.

## Materials and methods

Sequencing of the pol gene was carried out using Trugene genotyping kits, (Bayer diagnostics) in plasma samples from 10 recently diagnosed adults, average age 28.9±5.2 years, parenterally HIV infected. The nucleotide sequences were submitted to the Stanford database, and all strains were found to belong to the F subtype.

## Results

All patients had clinical progression, median CD4 count was 143.6cells/mmc (range 8-490); median HIV ARN was 380278.5copies/ml plasma (range38000-1020000) and they need to initialize antiretroviral treatment. We found PI major resistance mutations in 2 patients: the mutations were V82S, V82 F, I84L; NRTI resistance mutations in 7 patients, the mutations were: M41L, A62P, D67G, F77L, Q151R, K219P, M41V, T215N, K219Q, D67A, T69N, K70R, T215I, K219N and NNRTI resistance mutations in 3 patients, the mutations were: L100S, K103N, F227L, V179D, Y181S, . All patients presented other mutations in average number of 11.5±2 for protease and 26.7±19.8 for reverse transcriptase. From the type 2 TAMs recognized to confer NRTI drug resistance only K70R was found in 2 patients also K103N associated with primary NNRTI resistance was found in one patient. The L89M polymorphism, the most prevalent signature among treatment-naïve non-subtype B isolates, was found in 7 patients.

## Conclusions

Due to transmitted drug resistance only 3 of the tested patients could have been treated with any kind of antiretroviral therapy, the rest, 70%, have limited therapy option. If genotyping is not performed prior choosing an antiretroviral combination the chances of therapy efficacy are low.

